# Specific Abortive Treatment of Pneumonia

**Published:** 1892-01

**Authors:** 


### SPECIFIC ABORTIVE TREATMENT OF PNEUMONIA.

Francisco Moliner (*El Singlo Medico*, Madrid, October 18, 1891,) in an address before the Medical Congress of Valencia, insists that a specific abortive treatment of pneumonia is rational and possible. The idea of a morbific germ carries with it the idea of a specific remedy. Anything that will retard or cut short the evolution of the micro-organism will retard or cut short the clinical evolution of the disease. It is not necessary to kill the germ, but only to modify, in some degree, its virulence. Nor is it necessary that the remedy act on the microbe itself; it is enough if it act on the organism, increasing its resisting power, combating predisposition, or neutralizing the poisons generated. The rapidity of the development of the pneumococcus, indicates the necessity that the treatment should be begun early. One of the first pathoogical effects manifested in pneumonia is the alveolar congestion. This is caused by a ptomaine generated by the microbe which paralyzes the vaso-motors, and this congestion aids the further development of the micro-organism. Cold, acid media, well-oxygenated air and antiseptics, all retard their evolution. Hence the following plan of treatment is suggested: (1) The continued application of ice to the chest-walls, with inspiration of cold air. This hinders directly the growth of the germs, reduces the temperature, and combats the primary congestion. Lees gives statistics of sixteen cases treated in this way, with favorable results. (2) Inhalation of cold, oxygenated air impregnated with essential balsams. (3) Inhalations of sprays of acetic or lactic acids, of vapor of hydrofluoric acid, and rectal injections of hydro-sulphuric acid gas. At the same time give moderate doses of alcohol, which, acted on by the inhaled oxygen, liberates acetic acid in the blood. This treatment, to be effective, must be applied during the first forty-eight honrs after the initial chill, before the process has become generalized.—*University Medical Magazine.*

